# Epidemiology of Antibiotic Use and Drivers of Cross-Border Procurement in a Mexican American Border Community

**DOI:** 10.3389/fpubh.2022.832266

**Published:** 2022-03-10

**Authors:** Heather T. Essigmann, David A. Aguilar, William B. Perkison, Katherine G. Bay, Magdalena R. Deaton, Sharon A. Brown, Craig L. Hanis, Eric L. Brown

**Affiliations:** ^1^Division of Epidemiology, Human Genetics, and Environmental Sciences, Center for Infectious Disease, University of Texas Health Science Center, Houston, TX, United States; ^2^Division of Cardiovascular Medicine, University of Kentucky, Lexington, KY, United States; ^3^Division of Epidemiology, Human Genetics, and Environmental Sciences, Human Genetics Center, University of Texas Health Science Center at Houston, Houston, TX, United States; ^4^School of Nursing, University of Texas at Austin, Austin, TX, United States

**Keywords:** antibiotics, border health, acculturation, U.S.-Mexico border, health care, insurance, socioeconomic status

## Abstract

**Background:**

The U.S.-Mexico Border is an area of opportunity for improved health care access; however, gaps remain as to how and where U.S. border residents, particularly those who are underinsured, obtain care. Antibiotics are one of the most common reported drivers of cross-border healthcare access and a medication of particular concern since indiscriminate or inappropriate use is associated with antimicrobial resistance. In addition, many studies assessing preferences for Mexican pharmaceuticals and healthcare in U.S. border residents were done prior to 2010 when many prescription medications, including antibiotics, were available over the counter in Mexico.

**Methods:**

Data used in this study were collected during the baseline examination of an ongoing longitudinal cohort study in Starr Country, Texas, one of 14 counties on the Texas-Mexico border. Participants self-reported the name, date of use, and the source country of each antibiotic used in the past 12 months. Logistic regression was used to determine social, cultural, and clinical features associated with cross-border procurement of antibiotics.

**Results:**

Over 10% of the study cohort reported using antibiotics in the past 30 days with over 60% of all rounds used in the past 12 months sourced from Mexico. A lack of health insurance and generation score, a measure of acculturation, were the strongest predictors of cross-border procurement of antibiotics.

**Conclusions:**

Factors previously associated with cross-border acquisition of antibiotics are still present despite changes in 2010 to prescription drug regulations in Mexico. These results may be used to inform future public health initiatives to provide culturally sensitive education about responsible antibiotic stewardship and to address barriers to U.S. healthcare and pharmaceutical access in medically underserved, impoverished U.S.-Mexico border communities.

## Introduction

Nearly 2,000 miles long and spanning 62 miles north-to-south, the border between the United States (U.S.) and Mexico encompasses 80 Mexican municipalities and 44 U.S. counties with approximately 15 million people in residence (1, 2). Although governed by two independent political and legal systems with different health care systems and policies, these demographically and culturally analogous populations experience similar socioeconomic challenges as the region is consistently burdened by a lack of health insurance, high rates of unemployment, and poverty (1, 3). The socioeconomic status of U.S. border residents is disproportionately lower than their respective state populations and the U.S. average (4) such that if the 23 counties contiguous with the border formed a “51st state” (excluding the disproportionately affluent San Diego County) it would have the lowest proportion of residents with a high school education and rank last in per capita income and primary health care accessibility (2, 5).

Due to the lack of access to quality healthcare along the South Texas border, many border residents in both the U.S. and Mexico turn to informal healthcare options for the treatment of respective medical issues. Informal healthcare includes buying medications without prescription, using drugs prescribed to friends or family members, not claiming all of their earnings so that Medicaid can cover their children's medical needs, and seeking folk healing practices and practitioners (3). Therefore, healthcare providers and policymakers alike have long established that the U.S.-Mexico Border is an area of opportunity for improved health care access; however, gaps remain as to where and how U.S. border residents, particularly those who are uninsured or underinsured, obtain care (3). The U.S. Bureau of Transportation Statistics reported over 275 million crossings in 2019 alone (2) with prior studies indicating a high proportion of northbound crossings are U.S. residents returning from medical treatment or the purchase of medications in Mexican pharmacies (6–9).

The decision by U.S. residents to preferentially seek healthcare and pharmaceuticals in Mexico is influenced by several socioeconomic and acculturation-related factors. Financial barriers, including the absence of health insurance and high out-of-pocket costs, impede easy access to healthcare and prescription drugs in the U.S. and incentivize individuals in border communities to capitalize on the less expensive and more accessible alternatives in Mexico (3, 10). Mexican immigrants who live within close proximity to the U.S.-Mexico border and those who have lived in the United States for shorter periods of time are significantly more likely to seek medical and dental care in Mexico than their U.S. counterparts (9, 10) and U.S. residents of Mexican descent often report preference for cross-border health care based on shared cultural practices and language with their providers (6, 8, 9, 11–15).

Antibiotics are one of the most common reported drivers of cross-border healthcare access; a study of El Paso residents who reported crossing the border for medications found that 79% had purchased antibiotics at a Mexican pharmacy (6, 16). Cross-border procurement of antibiotics is of particular concern since indiscriminate or inappropriate use (i.e.,: taking an antibiotic for a viral or fungal infection or for an inappropriate duration) are drivers of antimicrobial resistance (17, 18), a global health problem that in the U.S. alone is responsible for 35,000 deaths a year with associated costs of 20 billion USD (17). Furthermore, suboptimal healthcare delivery and distribution systems in combination with access to inexpensive antibiotics may facilitate an environment primed for self-medicating behaviors without proper knowledge of their intended use (6, 13, 19, 20) and international differences in the licensing and regulation of pharmaceuticals may further impact quality of care and health outcomes for those crossing the border for antibiotics (19, 21, 22).

The Diabetes Prevention and Microbiome study is an ongoing longitudinal cohort initiated in 2017 designed to explore the role of the composition and function of the gut microbiome in the development and progression of type 2 diabetes in Mexican American adults residing in Starr County, Texas, one of 14 Texas counties on the U.S.-Mexico border. With a median household income of $29,294 in 2019, Starr County is among the most impoverished counties in the U.S. with over one-third of the 64,633 residents, 96% of whom self-identify as Hispanic, living below the federal poverty level (23). The prevalence of chronic disease, specifically type 2 diabetes mellitus and obesity, exceeds U.S. averages (24) and the county ranks in the bottom 20% for primary health care accessibility in Texas, hosting only 16 primary health care providers in 2019 (ranked 191 of 215 Texas counties surveyed) (25).

Given the economic challenges, low proportion of insured residents, and proximity to less expensive healthcare and pharmaceuticals in Mexico, Starr County can be leveraged to identify factors associated with cross-border antibiotic acquisition by individuals living along the U.S.-Mexico border. We hypothesize that those preferentially sourcing antibiotics in Mexico will differ by measures of acculturation and sociodemographic factors compared to those preferentially sourcing antibiotics from the United States. Understanding the forces driving the decision to obtain antibiotics in Mexico or the United States may help to both tailor culturally sensitive policies to improve healthcare access and develop educational tools to promote health, health literacy, and the importance of proper antibiotic usage and the consequences of misuse.

## Methods

### Participants

Data used in this study were collected during the baseline examination of the Diabetes Prevention and Microbiome study (DK116378), an ongoing longitudinal cohort of 616 participants designed to assess the relationship between the gut microbiome and type 2 diabetes. Baseline enrollment began on March 26th, 2018 and continued through March 16th, 2020. Subjects with prediabetes were recruited via separate funding (DK109920) to a diabetes prevention program based on Diabetes Prevention Program protocols (26) and previous diabetes education experience in this community (27–29). All remaining individuals were recruited from participants previously consented for re-contact, primarily from those individuals in a previous study exploring risk factors for type 2 diabetes (24). Informed consent was obtained from each participant in accordance with the Institutional Review Board of the University of Texas Health Science Center (HSC-SPH-06-0225) and the University of Texas at Austin (IRB #2016120040). Participants were enrolled, consented, and all questionnaires and medical exams were completed via interview by a trained bilingual field team at our field office established in 1981 in Rio Grande City, the County Seat of Starr County. As a result of the SARS-CoV-2 (“COVID19”) pandemic, there was a change to phone interviews for some follow-up exams, but these protocol changes did not alter questions administered to participants for the present study.

### Anthropometric and Clinical Values

Body weight was measured on a Tanita Total Body Composition Analyzer (TBF-400, Arlington Heights, Illinois) with individuals in street clothing and no shoes. Height was obtained using a wall-mounted stadiometer. Body mass index (BMI) was calculated by dividing weight in kilograms by the square of height in meters. Traditional, clinical cut-points of BMI were used in analyses (NIH, 1998). Following a blood draw, fasting glucose levels were determined on-site, in duplicate, using an YSI 2300 STAT Plus Glucose and Lactate Analyzer (YSI Life Sciences, Yellow Springs, Ohio).

### Sociodemographic Data

Information on education, income, employment, and health insurance was collected at the first post-baseline examination using questionnaires designed to assess socioeconomic status in the study population. Years of education were self-reported and recorded continuously as the number of completed years of education. Participants reported the total household income earned by all individuals living in the household by selecting the category that best described their situation ( ≤ $20,000, $20,001–30,000, $30,001–40,000, $40,001–50,000, $50,001–75,000, $75,000–100,000, or >$100,000). Health insurance status was collected by asking participants “Do you currently have health insurance?” with three possible response categories: “I have insurance through my employment,” “I have insurance through Medicare or self” or “I am not insured.” With respect to employment status, participants were asked to select whether they were “working full time,” “working part time,” “unemployed,” “retired,” “on extended sick leave,” or “disabled.”

### Acculturation Data

Acculturation data were collected during the baseline exam. Language, media, and social acculturation was assessed via the Short Acculturation Scale for Hispanics (SASH) developed by Marin et al. (30). Briefly, the SASH questionnaire contains 10 questions of equal weight (five assessing language acculturation, one concerning media acculturation, and four assessing social acculturation) each of which is scored on a Likert-type scale ranging from one to five with lower scores reflecting preferences for Spanish-speaking interactions or Hispanic/Latino social interactions or entertainment. Total acculturation score was the sum of the language, media, and social acculturation scores.

Cultural food preferences were assessed by asking participants “Of Hispanic/Latino and American food, do you usually eat…?” and allowing them to select which of four categories, ranging from “Mainly Hispanic/Latino foods” to “Mainly American foods,” best described their diet.

Generation score was used as parsimonious method to assess the effect of ancestry of the participants, their parents, and their grandparents (31). The generation score is a single continuous measure ranging from zero (participant and all parents and grandparents foreign-born) to 12 (participant and all parents and grandparents US-born) that succinctly reflects the variability of subjects, their parents' and grandparents' ancestry. The score is calculated by allotting four points to each generation born in the US, such that four points are awarded to a subject born in the US, 2 points are awarded for each parent, and 1 point for each parent bornin the US (31). Participants were also asked to self-report the number of years lived in Starr County, Texas, as well as the age they moved to the county.

### Antibiotic Procurement and Use

At baseline examination (baseline examinations occurred between March 2018 and March 2020), participants self-reported the name of each antibiotic used in the past 12 months, the date of use, and the country from which each antibiotic was sourced. For those that could only report month and year of use, date of most recent use was assumed to be the first of the month. Antibiotics were subsequently categorized into broad classes (i.e., penicillin, cephalosporin, others) based on their mode of action. Antivirals and antifungals were reported but were excluded from analysis.

For analyses of antibiotic source, participants were considered users of antibiotics if they reported the use of antibiotics at any time within the 12 months prior to the baseline exam. Users of multiple rounds of antibiotics that sourced their antibiotics from both the U.S. and Mexico were assigned a preferred source based on where the majority of their antibiotics were sourced (e.g., a person that reported taking four separate antibiotics, three sourced from the U.S. and one from Mexico respectively, would be classified as preferring to source their antibiotics from the U.S.). If participants reported sourcing equal numbers of antibiotics from the U.S. and Mexico, they were classified by determining the predominant source country for all prescription medications they reported taking at baseline.

### Statistical Analysis

Descriptive statistics of baseline characteristics were assessed using mean and standard deviations for continuous measures and counts and percent for categorial variables. χ^2^ and Mann-Whitney *U*-tests were used to compare antibiotic users to non-users for categorical and continuous variables, respectively. Univariate logistic regression was used to compare baseline characteristics by preferred antibiotic source location (U.S. or Mexico). Purposeful variable selection was used to build a multivariable model; variables with a univariable likelihood ratio *p*-value < 0.20 were considered sufficiently associated with preferred antibiotic source and were entered in the multivariable model. Covariates with a likelihood ratio *p*-value of < 0.10 in the multivariable model were retained in the final model. Correlations between categorical predictors were determined using Pearson's χ^2^ and Spearman's rho correlation coefficient for categorical and continuous predictors, respectively, and linearity assumptions were tested by comparing the log likelihood, and Akaike and Bayesian Information Criterion of a model with the linear predictor against one using restricted cubic splines and locally weighted scatterplot smoothing (Lowess plots). Receiver operator characteristic (ROC) curves were used to assess model efficacy. A two-sided *p*-value < 0.05 was considered statistically significant in all models. All analyses were performed using STATA 16 (Statacorp, College Station, Texas).

## Results

### Baseline Cohort

Six hundred and sixteen adults (28.73% male) completed their baseline examination prior to February 2020. Missingness ranged from 0 to 1.6%. Missing data were not imputed. The mean age of participants at baseline was 50.01 years and ranged from 35 to 69 years (Table 1). 52.60% of the participants were uninsured and 24.03% were unemployed. The majority (70.29%) were born in Mexico as were their parents and grandparents (mean generation score = 2.41, standard deviation = 3.81), but nearly 95% reported living in Starr County, Texas for more than a quarter of their lives. Over 40% of participants (41.52%) reported a combined household income of ≤ $20,000 in the past 12 months. Measures of language, media, and social acculturation collected using the Short Acculturation Scale for Hispanics (SASH) suggested a preference for speaking Spanish and Hispanic/Latino social interactions, but the majority (66.23%) reported no preference between Latino/Hispanic and American foods.

**Table 1:** Descriptive statistics of the baseline cohort stratified by any antibiotic use in the past 12 months.

**Characteristic**	**All participants (*n* = 616)**	**Antibiotic non-users (*n* = 342)**	**Antibiotic users (*n* = 274)**	***p*** -value^*^
	**Mean (SD) or *N* (%)^**∧**^**	**Mean (SD) or *N* (%)^**∧**^**		
**Anthropometric and clinical characteristics**				
Age at baseline (years)	50.01 (8.08)	50.27 (7.95)	49.69 (8.23)	0.35
Gender				**<0.001**
Male	177 (28.73)	118 (34.50)	59 (21.53)	
Female	439 (71.27)	224 (65.50)	215 (78.47)	
Fasting glucose at baseline (mg/dL)	105.05 (24.51)	106.61 (27.81)	103.11 (19.50)	0.06
BMI at baseline (kg/m^2^)				0.80
18.5–24.99	64 (10.39)	28 (8.19)	36 (13.14)	
25–29.9	183 (29.71)	109 (31.87)	74 (27.01)	
30–39.99	302 (49.03)	173 (50.58)	129 (47.08)	
>40.0	67 (10.88)	32 (9.36)	35 (12.77)	
**Sociodemographics**				
Number of years of education completed	10.01 (3.59)	9.74 (3.71)	10.36 (3.41)	**0.03**
Household income in past 12 months				0.80
≤ $20,000	252 (41.52)	147 (42.98)	105 (38.32)	
$20,001–30,000	139 (22.90)	74 (21.64)	65 (23.72)	
$30,001–40,000	58 (9.56)	31 (9.06)	27 (9.85)	
$40,001–50,000	58 (9.56)	28 (8.19)	30 (10.95)	
$50,001–75,000	56 (9.23)	30 (8.77)	26 (9.49)	
>$75,000	44 (7.25)	27 (7.89)	17 (6.20)	
Insurance status				0.90
Insured	282 (45.78)	155 (45.32)	127 (46.35)	
Not insured	324 (52.60)	182 (53.22)	142 (51.82)	
Employment status				0.99
Full time	276 (44.81)	155 (45.32)	121 (44.16)	
Part time	123 (19.97)	69 (20.18)	54 (19.71)	
Unemployed	148 (24.03)	81 (23.68)	67 (24.45)	
Retired/leave/disabled	60 (9.74)	32 (9.36)	28 (10.22)	
Marital status				0.45
Married	431 (69.97)	244 (71.35)	187 (68.25)	
Never married	65 (10.55)	31 (9.06)	34 (12.41)	
Divorced or separated	92 (14.94)	52 (15.20)	40 (14.60)	
Widowed	27 (4.38)	14 (4.09)	13 (4.74)	
**Acculturation**				
Generation score	2.41 (3.81)	2.44 (3.89)	2.39 (3.72)	0.86
Total acculturation score	18.65 (7.19)	18.35 (7.33)	19.01 (7.00)	0.08
Language acculturation score	8.69 (4.84)	8.50 (4.86)	8.91 (4.82)	0.10
Social acculturation score	7.68 (2.28)	7.64 (2.40)	7.73 (2.14)	0.41
Media acculturation score	2.28 (1.49)	2.21 (1.46)	2.37 (1.52)	0.18
Food preference				0.44
Mainly Latino foods	92 (14.94)	58 (16.96)	34 (12.41)	
Mostly Latino/Some American foods	106 (17.21)	56 (16.37)	50 (18.25)	
Equal Latino and American foods	408 (66.23)	222 (64.91)	186 (67.88)	
Mostly American foods	10 (1.62)	6 (1.75)	4 (1.46)	
Age moved to Starr County (years)	17.63 (13.00)	18.56 (13.43)	16.48 (12.32)	0.09
Years lived in Starr County	32.35 (12.40)	31.68 (12.59)	33.20 (12.14)	0.23
% of life lived in Starr county				0.22
0–25%	31 (5.03)	22 (6.43)	9 (3.28)	
25–50%	149 (24.19)	87 (25.44)	62 (22.63)	
50–75%	217 (35.23)	114 (33.33)	103 (37.59)	
75–100%	219 (35.55)	119 (34.80)	100 (36.50)	

* *Chi-squared test for categorical variables; Mann-Whitney U-test for continuous variables*.

∧ *Some values do not sum to 100% due to missingness*.

*The bold values are those that have asterisks or are highlighting those values that are of statistical significance*.

Two hundred and seventy-four subjects (44.48%) reported taking one or more antibiotics (range: 1–9 antibiotics) in the previous 12 months, 63.5% (174/274) of whom preferred to source them from Mexico. Of the 455 rounds of antibiotics used, two rounds for two separate participants were missing a source country; however, both participants reported acquiring 100% of their medications other than antibiotics from the U.S. and were consequently classified as preferred users of antibiotics purchased in the U.S. Most antibiotic users (260/274; 94.89%) reported using antibiotics exclusively purchased from either the U.S. (90/274; 32.85%) or Mexico (170/274; 62.04%). Of the 12 participants who sourced rounds of antibiotics from both sources, seven (7/12; 58.33%) showed preference for the U.S. and three (3/12; 25.00%) for Mexico and were classified accordingly. For the two remaining participants (2/12; 16.66%) that sourced antibiotics from both countries in equal proportions, their preferred source country was determined using the predominant source country for all prescription medications reported at baseline, resulting in 1 each categorized as a preferred user of antibiotics purchased in the U.S. and Mexico, respectively.

Sixty-five participants (10.55%) took at least one antibiotic during the 30 days prior to their baseline exam, 72.31% (47/65) of whom sourced the most recent course from Mexico. 36.86% (101/274) of users reported consuming two or more rounds in the past year (range 2–9 rounds) resulting in an estimate of 738.64 rounds of antibiotics/1,000 people (Table 2). Penicillin-class antibiotics were the most used class of antibiotic (51.87% of all rounds), nearly 80% of which were sourced in Mexico, followed by cephalosporins (8.79%), quinolones (7.69%), marcrolides (6.59%), and folate synthesis inhibitors (2.86%). Nitrofurans, nitromidazoles, and tetracyclines were each used by fewer than 1.5% of participants, excluding those who could not recall the antibiotic used (18.90%). Antibiotics of unknown class were equally likely to be sourced in the U.S. and Mexico.

**Table 2:** Number of respective antibiotic classes used in the 12 months prior to baseline by source country.

**Antibiotic class**	**Total rounds (*n* = 455) *N* (% of total)**	**Sourced from Mexico (*n* = 275) *N* (% of class)**	**Sourced from the U.S. (*n* = 180) *N* (% of class)**
Penicillin	236 (51.87)	187 (79.24)	49 (20.76)
Cephalosporin	40 (8.79)	13 (32.50)	27 (67.50)
Quinolone	35 (7.69)	12 (34.29)	23 (65.71)
Macrolide	30 (6.59)	8 (26.67)	22 (73.33)
Folate synthesis inhibitor	13 (2.86)	7 (53.85)	6 (46.15)
Nitrofuran	6 (1.32)	3 (50.00)	3 (50.00)
Tetracycline	5 (1.10)	1 (20.00)	4 (80.00)
Nitroimidazole	3 (0.66)	0 (0.00)	3 (100.00)
Ophthalmic ointment	1 (0.22)	1 (100.00)	0 (0.00)
Unknown	86 (18.90)	43 (50.00)	43 (50.00)

### Antibiotic Users vs. Non-users

Characteristics of study participants stratified by antibiotic use in the past 12 months are also described in Table 1. Females were significantly more likely than males to use antibiotics (48.9% of females and 33.3 % of males used antibiotics; *p*-value < 0.001), and to have completed more years of education (*p*-value = 0.02). Lower fasting blood glucose was a marginally significant predictor of antibiotic use. Users and non-users of antibiotics were not statistically different with respect to household income in the past year, insurance employment, marital status, or measures of acculturation (e.g.,: generation score, years residence in or age moved to Starr County, or SASH acculturation measures).

### Univariable Comparisons of Antibiotic Users by Preferred Source Country

Univariate logistic regression was used to determine potential clinical, socioeconomic, or acculturation predictors of preferred antibiotic source location (U.S. or Mexico) (Table 3) for those that reported the use of antibiotics within the previous 12 months. There were no differences by age, gender, or other clinical or anthropometric variables of interest by preferred source of antibiotic procurement.

**Table 3:** Univariable logistic regression: odds of preferentially sourcing antibiotics from Mexico.

	**Primary source of antibiotics Mean (SD) or** ***N*** **(%)**^**∧**^		
**Predictors**	**US (*n* = 100)**	**Mexico (*n* = 174)**	**Odds ratio (95% CI)**
**Anthropometric and clinical characteristics**			
Age at baseline (years)	49.82 (8.43)	49.61 (8.13)	1.0 (0.97–1.03)
**Gender**			
Male	24 (24.00)	35 (20.11)	**REF**
Female	76 (76.00)	139 (79.89)	1.25 (0.70–2.26)
Fasting glucose at baseline (mg/dL)	105.38 (24.11)	101.80 (16.21)	0.99 (0.98–1.00)
**BMI at baseline (kg/m** ^**2**^ **)**			
18.5–24.99	14 (14.00)	22 (12.64)	**REF**
25–29.9	27 (27.00)	47 (27.01)	1.11 (0.49–2.52)
30–39.99	45 (45.00)	84 (48.28)	1.19 (0.55–2.54)
>40.0	14 (14.00)	21 (12.07)	0.95 (0.37–2.47)
Rounds of antibiotics (1–4+)^∧∧^	1.87 (1.47)	1.54 (0.89)	0.80 (0.61–1.04)
**Sociodemographics**			
**Education**			
0–8 years	26 (26.00)	50 (28.74)	**REF**
9–12 years	44 (44.00)	90 (51.72)	1.06 (0.59–1.93)
13 or more years	28 (28.00)	30 (17.24)	0.56 (0.28–1.12)
**Household income in past 12 months**			
≤ $20,000	38 (38.00)	67 (38.51)	**REF**
$20,001–30,000	26 (26.00)	39 (22.41)	0.85 (0.45–1.61)
$30,001–40,000	16 (16.00)	41 (23.56)	1.98 (0.74–5.35)
$40,001–50,000	10 (10.00)	20 (11.49)	1.13 (0.48–2.67)
$50,001–75,000	9 (9.00)	17 (9.77)	1.107 (0.44–2.64)
>$75,000	9 (9.00)	25 (14.37)	0.50 (0.18–1.42)
**Insurance status**			
Insured	64 (64.00)	63 (36.21)	**REF**
Not Insured	33 (33.00)	109 (62.64)	3.36 (1.99–5.66)^***^
**Employment status**			
Full time	47 (47.00)	74 (42.53)	**REF**
Part time	14 (14.00)	40 (22.99)	1.81 (0.89–3.69)
Unemployed	18 (18.00)	49 (28.16)	1.72 (0.90–3.31)
Retired/leave/disabled	19 (19.00)	9 (5.17)	0.30 (0.13–0.72)^**^
**Marital status**			
Married	13 (13.00)	21 (12.07)	**REF**
Never married	63 (63.00)	124 (71.26)	0.82 (0.39–1.75)
Divorced or separated	19 (19.00)	21 (12.07)	0.56 (0.28–1.12)
Widowed	5 (5.00)	8 (4.60)	0.81 (0.26–2.59)
**Acculturation**			
Generation score	3.66 (4.42)	1.66 (3.03)	0.87 (0.81-0.93)^***^
Total acculturation score	21.12 (7.30)	17.80 (6.54)	0.93 (0.90–0.97)^***^
Language acculturation subscore	10.13 (5.08)	8.21 (4.54)	0.92 (0.88–0.97)^**^
Social acculturation subscore	8.22 (2.28)	7.45 (2.01)	0.84 (0.75–0.95)^**^
Media acculturation subscore	2.77 (1.59)	2.14 (1.43)	0.76 (0.65–0.90)^***^
**Food preference**			
Mainly Latino Foods	8 (8.00)	26 (14.94)	**REF**
Mostly Latino/Some American Foods	19 (19.00)	31 (17.82)	0.50 (0.19–1.33)
Equal Amounts or Mostly American Foods^∧∧^	73 (73.00)	117 (67.24)	0.49 (0.21–1.14)
Age moved to Starr County (years)^#^	13.97 (12.64)	17.93 (11.94)	1.14 (1.03–1.25)^*^
Years lived in Starr County^#^	35.85 (12.16)	31.68 (11.93)	0.83 (0.76–0.92)^**^
**% of life lived in Starr county**			
0–50%^∧∧^	19 (19.00)	52 (29.89)	**REF**
50–75%	34 (34.00)	69 (39.66)	0.74 (0.38–1.44)
75–100%	47 (47.00)	53 (30.46)	0.41 (0.21–0.79)^**^

* *Wald p-value ≤ 0.05;*

** *≤ 0.01;*

*** *≤ 0.001*.

∧∧ *Categories collapsed to avoid small cells*.

∧ *Some values do not sum to 100% due to missingness*.

# *OR interpreted as a 5-year increase*.

*The bold values are those that have asterisks or are highlighting those values that are of statistical significance*.

Health insurance status was strongly associated with preferred source country for antibiotics. Those without insurance had 3.36 times the odds (95% CI: 1.99–5.66) of acquiring their antibiotics in Mexico compared to those with health insurance. Those with part-time or no employment were more likely to cross the border for their antibiotics than those with full-time employment, but the effect was not statistically significant; however, those that were retired, on leave, or disabled were significantly less likely to cross the border for antibiotics (OR = 0.30; 95% CI: 0.13–0.72). Despite associations with both employment and insurance status, the antibiotic source was not significantly associated with household income over the past year nor was marital status or education.

While no single factor was associated more strongly with preferring one source country for antibiotics as was health insurance status, nearly all acculturation variables were associated with preferentially acquiring drugs from one country. A one-unit increase in generation score, reflecting a greater proportion of U.S.-born ancestry, was associated with a 13% decrease in the odds of preferentially sourcing antibiotics in Mexico (OR = 0.87; 95% CI: 0.81–0.93).

The total SASH acculturation score and each of the sub scores (language, media, and social acculturation) were significantly associated with preferred antibiotic source. Overall, a one-point increase in total acculturation score resulted in a 7% decrease in the odds of sourcing antibiotics from Mexico (OR = 0.93; 95% CI: 0.90–0.97). Each one unit increase in language score, equivalent to a one unit increase in preference for speaking English, was associated with an 8% decrease in the odds of attaining antibiotics in across the border (OR = 0.92; 95% CI: 0.88–0.97). Similarly, for each one unit increase in media and social acculturation scores, indicating a lower preference for Hispanic or Latino media and social interactions, there was a 24% (OR = 0.76; 95% CI:0.65–0.90) and 16% (OR = 0.84; 95% CI: 0.75–0.95) reduction in the odds of buying antibiotics from Mexico, respectively.

Both the length of time living in Starr County and the participant's age when they moved to the county was associated with antibiotic source preference. For each additional 5 years that a participant lived in Starr County, there was 17% reduction in the odds (OR = 0.83; 95% CI: 0.76–0.92) of crossing the border into Mexico for antibiotics, and each 5 years older a person was upon moving to the county meant a 14% increase in the odds of the same (OR = 1.14; 95% CI: 1.03–1.25). Similarly, those that lived at least 75% of their life in the county were over 50% less like to cross the border to buy antibiotics compared to those that resided in the area for less than half their life (OR = 0.41; 95% CI: 0.21–0.79).

### Multivariable Comparisons Between Users of Antibiotics by Preferred Source Country

To avoid multicollinearity, generation score and the total SASH acculturation score were used to assess the effects of acculturation in the multivariable model. In the final model, health insurance status [adjusted OR (aOR) = 3.18; 95% CI: 1.86–5.45], generation score [adjusted OR (aOR) = 0.87; 95% CI: 0.81–0.93], and number of rounds of antibiotics used in the past year (aOR = 0.75; 95% CI: 0.56–1.00) were the only variables selected for inclusion and each changed only minimally from univariable modeling, with all but the later as significant predictors of preferred antibiotic sourcing (Table 4). The area under the ROC curve for this model was 0.72 suggesting that these three factors alone may have good discriminatory performance to determine the country from which study subjects were likely to buy their antibiotics.

**Table 4:** Multivariable logistic regression: odds of preferentially sourcing antibiotics from Mexico.

**Predictors**	**Odds ratio (95% CI)**
Generation score	0.87 (0.81–0.93)^***^
Rounds of antibiotics used (1 to 4+ rounds)	0.76 (0.57–1.02)
Insurance status	
Insured	**REF**
Not Insured	3.31 (1.93–5.70)^***^

*** *Wald p-value ≤ 0.001*.

*The bold values are those that have asterisks or are highlighting those values that are of statistical significance*.

Sensitivity analyses removing the 14 participants that sourced antibiotics from both the U.S. and Mexico or for whom the source was established through determination of preferred source of all prescription medication (data not shown) resulted in only minor changes to the final model: the effect of rounds of antibiotics decreased (aOR = 0.83; 95% CI: 0.60–1.14), generation score was essentially unchanged (aOR = 0.88; 95% CI: 0.81–0.94), and that of insurance increased (aOR = 4.02; 95% CI: 2.27–7.13).

## Discussion

We explored the epidemiology of antibiotic use in Mexican American residents of an economically disadvantaged and medically underserved U.S.-Mexico border community to better understand predictors affecting their choice of obtaining antibiotics whether formally or informally (i.e., obtaining antibiotics without prescription or from friends in family) in Mexico or the United States (3). In Starr County, antibiotics are also often purchased at flea markets (personal communication). The source population for this study was a community of Mexican Americans living in Starr County, Texas, which ranks in the bottom 20% of Texas counties for primary health care accessibility with only 24.8 primary care physicians for every 100,000 residents (the state's largest county, Harris county, by comparison has 88.0 physicians/100,000) (25). Over 50% of the study participants were uninsured and nearly a quarter were unemployed with 41.52% of participants reporting their total household income in the past 12 months was ≤ $20,000.

Nearly 45% of our study population reported using antibiotics in the past year with most (63.5%) preferring to source these prescriptions from Mexico. Penicillin-class drugs, nearly 80% of which were sourced in Mexico, made up over half of all antibiotics used, a prevalence considerably higher than previous national estimates of 31.9% and 27.5% (32, 33). Eighty percent of penicillins were obtained in Mexico and 20% from the US. The sourcing of the next 3 most commonly prescribed antibiotics was almost exactly opposite i.e., cephalosporins, quinolones, and macrolides were primarily obtained in the US (68, 66, and 73%, respectively; Table 2). This suggested that these less frequently known antibiotics were likely obtained by prescription in the US and the more-commonly known penicillin-based drugs obtained in Mexico, likely without a prescription. The estimated rate of total antibiotic use in this population, 738.64 rounds of antibiotics per 1,000 persons, fell within the middle of the wide range of prior estimates of outpatient antibiotic use in the U.S. (32–38), possibly reflecting factors unique to this population, including the limited geographic area sampled or the high unemployment and low insurance rates of participants in our study. The 30-day prevalence of antibiotic use in this population, however, was high i.e., 10.55% (65/616) of participants reported using antibiotics in the 30 days before their first exam, over 70% of whom reported purchasing their most recent course from Mexico. This 30-day prevalence is more than double a 2012 National Health and Nutrition Examination Survey (NHANES) estimate of 4.1% for use in the general U.S. population and 3.8% for Mexican Americans (39). This 30-day prevalence was stable in our study population with 9.8% of subjects reporting in antibiotic use in the 30 days prior to their second examination, 62.22% of which were sourced in Mexico.

We did not have data on whether the antibiotics used were taken under the guidance of a licensed provider, but the high 30-day prevalence raises concerns for antimicrobial resistance. Many studies assessing preferences for Mexican pharmaceuticals and healthcare in border residents were done prior to 2010 when many prescription medications, including antibiotics, were available over the counter in Mexico (40). Changes made that year prohibited the sale of antibiotics without a prescription from a licensed provider, yet there is evidence that permissive attitudes toward dispensing antibiotics without demonstration of sufficient need remain (7). Specifically, antibiotics are easily purchased without prescription in flea markets or obtained from friends or relatives, or leftover antibiotics from previous prescriptions are consumed (41–43). In both Mexico and the U.S., medical practitioners report feeling pressured by patients to provide antimicrobials where their use may not be warranted (7). Compounding the problem, prior research suggested that Mexican Americans have higher expectations than other consumers that healthcare providers prescribe antibiotics to treat a cough or a cold and are nearly three times more likely than other populations to report consuming antibiotics that were not prescribed by a health care practitioner (11, 44).

Although much of the literature associating socioeconomic and cultural determinants of health to antibiotic procurement in Mexico was undertaken when antibiotics were available over the counter in Mexico, many of our findings are consistent with previous studies associating lack of health insurance and measures of acculturation status toward Mexican or Hispanic culture or Spanish-language interactions with sourcing antibiotics in Mexico (11, 12, 44). Lack of health insurance status, generation score, and number or rounds of antibiotics used were the only predictors retained in the final multivariable model, but these three factors alone had good discriminatory performance to determine which country study subjects were likely to buy their antibiotics from (ROC area under the curve = 0.72).

The absence of health insurance coverage among Starr County residents was a significant predictor for the purchase of antibiotics in Mexico. Less than 50% of study participants reported health insurance coverage at baseline and those without coverage had over three times the odds of using antibiotics acquired from Mexico after adjusting for generation score and rounds of antibiotics used (aOR = 3.31; *p*-value < 0.001, Table 3). These findings are consistent with the 2015–2017 National Health Interview Survey that reported participants who were uninsured had three times the odds of seeking prescription medication outside the U.S compared to those who were insured (adjusted OR = 3.14; 95% CI: 2.33–4.21) (45). Numerous studies have repeatedly demonstrated that uninsured and underinsured border residents seek care in Mexico because it is less expensive and more accessible than care in the U.S. (7, 9, 12, 44, 46). The preference for Mexican antibiotics in our underinsured, low-income study cohort suggested that more affordable antibiotics across the border may provide an avenue for obtaining prescription drugs for those for whom care might otherwise be unaffordable in the U.S. It is likely that lack of insurance will remain a driver of cross-border health care since recent changes in immigration policy and the Affordable Care Act are projected to increase the number of uninsured Texans from 4.8 million in 2019 to 6.1 million over the next two decades (Texas Alliance for Health Care, 2019, http://wrgh.org/docs/The_Impact_of_Uninsurance_on_Texas_Economy_20190108.pdf). An expected 6.3% per year increase in the cost of prescription drugs through 2025 may further intensify pressure to seek alternative care outside the U.S. due to costs and accessibility (47). In addition, Hispanics and other minorities are less likely to benefit from medication therapy management programs as many do not qualify for Medicare or for the Patient Protection and Affordable Care Act among those with Medicare Part D (48).

Acculturation, a measure of assimilation into a culture, is known to dramatically impact both health and healthcare access in the U.S. In our study, each one-point increase in generation score, reflecting increased US-born ancestry and likely indicative of preferences for English language interactions and acculturation toward American culture, reduced the odds of purchasing antibiotics in Mexico by 13% (aOR: 0.87; *p*-value < 0.001) independent of other factors. These findings agree with previous associations between stronger connections with relatives from Mexico, familiarity with Mexican customs, recent immigration status, and Hispanic origins and cross-border healthcare access (6, 9, 45, 49, 50). There is evidence many Mexican Americans distrust American healthcare systems and providers and express greater confidence in care and drugs they receive in Mexico (11, 12). Although previous studies identified language preference to be a particularly important predictor of cross-border health care this does not appear to be the case in Starr County where all 16 healthcare providers speak Spanish, suggesting other factors influenced the purchase of antibiotics across the border (6). It should also be pointed out that with 96% identifying as Mexican American, Starr County is unusual compared to other border counties that are not this homogeneous. Given that English language proficiency is highly associated with health literacy, ability to navigate the U.S. health care system, and consequently with health status, it is not surprising that acculturation status remains an important predictor of cross-border healthcare utilization in border residents (20, 51).

This study has notable strengths, including a large number of participants in an established cohort with high participation rates that may be generalizable to U.S.-Texas border populations. Possible limitations include a lack of information regarding antibiotic doses, the reason antibiotics were taken, treatment durations, the point source of antibiotics (other than the country of origin), or whether the antibiotics were used under guidance of a licensed provider. To reduce selection bias, participants were not asked about their citizenship status; this, however, may be a predictor to better understanding drivers for cross-border antibiotic sourcing.

In summary, over 10% of our study cohort of U.S.-Mexico border residents reported using antibiotics in the past 30 days with over 60% of all rounds in the prior year preferentially sourced from Mexico. The primary drivers of preference for Mexican antibiotics were a lack of health insurance and greater acculturation toward Mexican culture, suggesting factors previously associated with cross-border acquisition of pharmaceuticals are still present despite changes in 2010 to prescription drug regulations in Mexico. Antibiotic stewardship programs in hospitals have been established to mitigate the ever-growing problem of antimicrobial resistance. However, no such programs, at the community level have been established (including educating the public regarding the importance of using these drugs wisely). These results may be used to inform future public health initiatives to provide culturally sensitive education about responsible antibiotic stewardship and to address barriers to U.S. healthcare and pharmaceutical access in medically underserved, impoverished U.S.-Mexico border communities.

## Data Availability Statement

The raw data supporting the conclusions of this article will be made available by the authors, without undue reservation.

## Ethics Statement

The studies involving human participants were reviewed and approved by Institutional Review Board of the University of Texas Health Science Center (HSC-SPH-06-0225) and the University of Texas at Austin (IRB #2016120040). The patients/participants provided their written informed consent to participate in this study.

## Author Contributions

EB, CH, and SB are the principal investigators and oversaw the study design and data collection. HE also participated in data collection and data analyses including statistical analyses. HE, WP, KB, MD, SB, CH, and EB participated in manuscript preparation. All authors have read and approved the final version of the manuscript.

## Funding

This work was supported by National Institutes of Health grants R01DK116378 to EB, R01DK116378 to SB and CH, and grant 5T42OH008421 from NIOSH/Centers for Disease Control and Prevention to WP.

## Conflict of Interest

The authors declare that the research was conducted in the absence of any commercial or financial relationships that could be construed as a potential conflict of interest.

## Publisher's Note

All claims expressed in this article are solely those of the authors and do not necessarily represent those of their affiliated organizations, or those of the publisher, the editors and the reviewers. Any product that may be evaluated in this article, or claim that may be made by its manufacturer, is not guaranteed or endorsed by the publisher.
